# Factors affecting the early failure of implants placed in a dental practice with a specialization in implantology – a retrospective study

**DOI:** 10.1186/s12903-019-0900-8

**Published:** 2019-09-05

**Authors:** Johannes Krisam, Larissa Ott, Stephanie Schmitz, Anna-Luisa Klotz, Aida Seyidaliyeva, Peter Rammelsberg, Andreas Zenthöfer

**Affiliations:** 1 Institute of Medical Biometry and Informatics, University of Heidelberg, Im Neuenheimer Feld 130, 69120 Heidelberg, Germany; 2 Dental School, Department of Prosthodontics, University of Heidelberg, Im Neuenheimer Feld 400, 69120 Heidelberg, Germany; 3 Praxis für Zahnmedizin Dr. Schmitz, Hauptstraße 13, 69434 Hirschhorn, Germany

**Keywords:** Implant, Osseointegration, Early failure, Practice-based

## Abstract

**Background:**

To evaluate early failure and possible risk factors for failure of dental implants placed under practice-based conditions.

**Methods:**

To clarify the research question, anonymized data from 106 patients with 186 dental implants were analyzed. The presence of sucessful healing (yes/no) at the time of incorporation of the final prosthesis was assessed. Mixed models were compiled for each target variable to enable estimation of the effects of patient-related and implant-related conditions on the risk of early implant failure.

**Results:**

Nine out of 186 implants (4.8%) placed in 106 participants failed before incorporation of the final prosthesis. The use of shorter implants (< 10 mm) and the need for augmentation procedures were associated with a greater risk of early implant failure. For shorter implants, the risk was 5.8 times greater than that for longer implants (*p* = 0.0230). Use of augmentation procedures increased the risk by a factor of 5.5 (*p* = 0.0174).

**Conclusions:**

Implants placed in the dental practice with a specialization in implantology heal successfully. The use of augmentation procedures and of implants shorter than 10 mm seems to be associated with a greater risk of early implant failure.

## Background

Although advanced methods of oral-health preservation are delaying tooth loss to later in life, the loss of teeth is still a major problem in aging societies worldwide [1]. Tooth loss can affect chewing function and dental esthetics and, therefore, oral-health-related quality of life [2, 3]. Dentists often have to select conventional tooth-supported, implant-supported, or combined tooth–implant-supported prosthetic treatments on the basis of clinical conditions and patients’ requirements. Implant-supported dental prostheses are now widely used for the replacement of one or more missing teeth [4, 5]. Moreover, the use of dental implants can often avoid the integration of unrestored adjacent teeth or the use of a removable prosthesis. Implant systems characterized by micro-rough surfaces and internal abutment connections result in successful healing [6, 7] and long-term clinical performance [8, 9]. Nonetheless, it should be remembered that early failure (no or inadequate osseointegration, i.e., intimate bone-to-implant connection before functional loading) can also occur. Early failures account for approximately 2–6% (%) of implants placed, and the incidence can be even higher for implants placed in specific risk populations (for example (e.g.), patients receiving zygoma implants after tumor surgery or radiotherapy and/or chemotherapy) [10–18]. Early loss of an implant is not, however, an acute rejection reaction; rather, it is a consequence of bacterial colonization of the implant surface, which results in the development of fibrous scar tissue between the implant surface and the surrounding bone. Factors identified as being associated with early implant loss are diverse, as is the definition of early loss (endpoints, e.g., abutment connection, occlusal loading, one year after placement, etc.) [10–18]. Several studies have attributed the absence of healing to the implant site (maxilla or mandible, anterior or posterior) [12, 15, 16], smoking [10–13, 18], and comorbidities such as periodontitis [13] and metabolic diseases [10–12]. Poor healing is also linked with poor quality and low quantity of bone [11, 12], which frequently results in the need for augmentation procedures [13, 15, 17], or in the selection of short implants [14, 16–18]. Preoperative antibiotics, in contrast, seem to be a protective factor for primary healing [19]. Information about the factors affecting the osseointegration of dental implants placed in clinical practice, i.e., in contrast with standardized institutional studies, is especially valuable for the dental practitioner. Information about the practice-based performance of dental implants is sparse, especially information that enables estimation of the likelihood of early failure. It might be assumed that the success of implants placed in dental practices and clinics could differ from that observed in academic centers because of different surgical procedures, specializations, or patient populations [20]. However, a study from a private practice of 20 patients fitted with 31 implants revealed only one implant (3%) loss before loading [21]. Other studies by private clinics reported initial failure of approximately 2–3% [13, 22], which is indicative of healing comparable to that observed in institutional studies. These studies were, however, performed at private and / or specialized referral institutions. The lack of osseointegration of modern dental-implant systems and possible factors affecting this in a practice-based setting have not been fully and definitively clarified. The purpose of this retrospective study was, therefore, to evaluate the early implant failure and possible risk factors for failure of dental implants placed in a dental practice of which one specialization is implantology.

## Methods

### Study setting

This retrospective study was performed with anonymized patient data from a dental practice. The owner of the practice was asked to provide data, for scientific purposes, from patients who had received one or more dental implants. All implant cases taking place between 2012 and 2017 (during which time a single implant system only was used) were anonymized on site. A database was created for analytical purposes by a doctorate student. The study protocol was evaluated and approved by the local ethics committee (no. S-248/2017). Only cases for which target variables were complete were included in the analyses.

### Target variables

Consecutive numbers were chosen for each patient (hereafter called “participant”) and implant placed. Sociodemographic (age in years, gender, smoking) and medical (number of diseases, number of permanent medications, diabetes mellitus, history of periodontitis) issues were recorded in the database (patient-related factors). For the variables smoking, diabetes, and history of periodontitis, only their presence or absence was noted and used in statistical analysis (each considered as yes/no). A more detailed breakdown (for example, of the number of cigarettes smoked or glycemic values) was not available. The following were also documented for each implant (implant-related factors): implant length and width (mm), implant site (anterior or posterior, maxilla or mandible), torque applied during insertion, need for augmentation procedures (sinus-floor elevation; all types of grafting, including bone spreading or bone splitting), and mode of healing (open: healing abutment with penetration through the mucous membrane; closed: implant cover screw below the mucous membrane, re-exposure of implants needed after healing period). The healing period (months) was defined as the time between implant surgery and functional loading with the final prosthesis.

### Treatment

All implants placed at the dental practice from 2012 to 2017 were modern bone-level implants (blueSky; Bredent Medical, Senden, Germany). Surgery was performed by two dentists who offered the complete range of dental treatments, including prosthetic dentistry. One of the participating dentists (D1) graduated in 2008 and passed a specialized course in dental implantology offered by the German Society of Oral Implantology (‘Curriculum Implantology’). When treating the patient sample, her professional experience thus ranged between four and 9 years. The other dentist (D2) passed their final exams in 2015. All implant treatments performed by D2 were supervised by D1. All implants were placed in accordance with the manufacturers’ instructions by use of conventional treatment procedures (surgical handpieces, sterile covers, etc.) in accordance with a well-defined standard protocol. For each implant, the insertion torque applied at the time of placement was measured and documented. The minimum torque required was 20 Newtoncentimeter (Ncm). Patients with a history of periodontal disease were required to have a stable periodontal condition after treatment for a minimum of 3 months. Successful healing was assumed when the final abutment and prosthesis were attached and occlusally loaded and no clinical abnormities (e.g., radiologically evaluated bone loss < 2 mm were observed for the implants. The moment of loading was selected by the dentists (e.g., extent of augmentative measures/primary stability). Abutments were attached to implants by applying a torque of 25 Ncm. Before initiation of prosthetic management (impressions, maxillomandibular relationship), implants were evaluated clinically (resonance on percussion, probing depths, pain, loosening) and/or radiologically (bone loss).

### Statistical analysis

The primary outcome, successful osseointegration/healing, was considered dichotomously (0 = yes; 1 = no, i.e., failed before attachment of the final prosthesis). Predictor variables were gender, implant length (< 10 mm = 1; ≥ 10 mm = 0), implant diameter (< 4 mm = 1; ≥ 4 mm = 0), implant site (anterior = 0; posterior = 1), and jaw (mandible = 0; maxilla = 1). Torque was categorized as follows: < 30 Ncm = 1; ≥ 30 Ncm = 0). The continuous predictor variables of age, number of permanent medications, and number of diseases were dichotomized by using the median as the cut-off.

Chi-squared tests were used for bivariate comparison of an association between successful osseointegration and the predictor variables. For each possible predictor of the dependent variable, osseointegration (0 = yes; 1 = no), a univariate, linear mixed model including the patient as a random factor was also fitted to take the clustered structure of the data into account (teeth nested within patients). Odds ratios were obtained from all models, with respective *p*-values and 95% confidence intervals (CI). If a model did not converge, a Firth-corrected logistic regression model was used instead to obtain estimates of odds ratios. *P*-values were interpreted descriptively and were regarded as indicative of significance if they were < 0.05. Statistical analysis was performed by use of SAS v 9.4 (SAS Institute; Cary, NC, USA).

## Results

### Study population

Two-hundred and six implants in 116 patients were placed at the dental practice during the years 2012–2017. Seven implants with a reduced diameter (2.8 mm) placed in three participants had to be excluded from analysis. This was because these implants had a different design (one-piece tissue-level implants with, e.g., a ball welded onto the implant), and a sub-group analysis of *n* = 7 seemed insufficient to achieve meaningful results. Seven other participants with 13 implants who had either not yet been fitted with their final prosthesis or did not wish to continue treatment at the practice (e.g., because of moving home) were also excluded from analysis. Thus, 186 complete datasets (*n* = 106 participants) were analyzed (response ~ 90% for number of implants and participants analyzed). The mean (standard deviation, SD) age of the participants was 60.6 (12.7) years; 58.5% were female. Approximately one fifth of the participants were smokers. Participants suffered from a mean number (SD) of 0.8 (0.9) diseases and took 1.4 (2.0) permanent medications. Approximately 10% suffered from diabetes and 26% had a history of periodontitis. The mean healing time for the implants before the final prosthesis was attached and loaded was 147.8 (81.9) days, 154.3 (79.9) and 137.8 (91.2) days in the maxilla and the mandible, respectively). Detailed information about the implants (length, diameter, site, etc.) is presented in Table 1.

**Table 1:** Results from bivariate analysis of implant outcome and dichotomized predictor variables (*n* = 186)

	Number of healed implants (%)	Number of failed implants (%)	*p*-value
Age (years)			
< 61	86 (93.5)	6 (6.5)	0.290
≥ 61	91 (95.7)	3 (3.1)	
Gender			
Female	103 (96.3)	4 (3.7)	0.416
Male	74 (93.7)	5 (6.3)	
Diseases			
< 1	80 (95.2)	4 (4.8)	0.965
≥ 1	97 (95.1)	5 (4.9)	
Medications			
< 1	78 (94.0)	5 (6.0)	0.499
≥ 1	99 (96.1)	4 (3.9)	
Smoking			
No	136 (95.8)	6 (4.2)	0.484
Yes	41 (93.2)	3 (6.8)	
Diabetes			
No	159 (95.2)	8 (4.8)	0.927
Yes	18 (94.7)	1 (5.3)	
Periodontitis history			
No	121 (95.3)	6 (4.7)	0.915
Yes	56 (94.9)	3 (5.1)	
Implant length (mm)			
< 10.0	14 (82.4)	3 (17.6)	**0.010**
≥ 10.0	163 (96.4)	6 (3.6)	
Implant diameter (mm)			
< 4.0	40 (95.2)	2 (4.8)	0.979
≥ 4.0	137 (95.1)	7 (4.9)	
Implant location			
Anterior	43 (100)	0 (0.0)	*0.092*
Posterior	134 (93.7)	9 (6.3)	
Jaw			
Maxilla	104 (92.9)	8 (7.1)	*0.072*
Mandible	73 (98.6)	1 (1.4)	
Healing method			
Open	39 (100)	0 (0.0)	.113
Closed	138 (93.9)	9 (6.1)	
Bone augmentation			
No	144 (97.3)	4 (2.7)	**0.007**
Yes	33 (86.8)	5 (13.2)	
Torque reached (Ncm)			
< 30	59 (95.2)	3 (4.8)	1.000
≥ 30	118 (95.2)	6 (4.8)	

*P*-values are based on the chi-squared test. Significant *p*-values are marked in bold. *P*-values for trends (*p* < 0.100) are marked in italics

### Bivariate evaluation of participant-related and implant-related characteristics and implant healing

Nine out of 186 (4.8%) of the implants placed were lost before prosthetic restoration because of failed osseointegration. Significantly more failures were observed for shorter (< 10 mm) implants than for implants ≥10 mm long (*p* = 0.010). Losses were also more frequently observed for implants placed after the use of augmentation procedures (*p* = 0.007). A substantial trend toward greater failure of implant osseointegration was observed for implants placed in the maxilla compared with in the mandible (*p* = 0.072) and for posterior implants compared with implants placed in anterior sites (*p* = 0.092). All such failures were observed in posterior implant sites, and eight out of nine early failures occurred in the maxilla. The insertion torque used at the time of placement of the failed implants ranged between 20 and 45 Ncm (mean: 32 Ncm). None of the other target variables, including smoking (failure rate: 6.8%; *p* = 0.484) and diabetes (failure rate: 5.3%; *p* = 0.977), reached a level of statistical significance or a statistical trend (*p* > 0.100). Detailed numbers of failures and comparisons of implant losses are presented in Table 1.

### Estimation of risk factors for early implant loss

The effects of augmentation procedures (*p* = 0*.*0174) and implant length (*p* = 0.023) on implant healing were reproduced when adjusted for the different number of implants per participant (Table 2). Univariate, mixed-model analysis revealed a 5.5-fold greater risk of failure of osseointegration (95% CI 1.4–21.7) for implants placed in an augmented implant site. A 5.8-fold greater risk was identified for short (< 10 mm) implants (95% CI 1.3–26.4).

**Table 2:** Results from univariate, mixed-model analysis with the dependent variable osseointegration (yes/no), participants as random factor, and the respective predictor as fixed factor (*n* = 186)

		95% CI		
Variable	Odds ratio	Lower limit	Upper limit	*p*-value
Older age	0.5	0.1	2.0	0.3029
Female	0.6	0.1	2.3	0.4232
More diseases	1.0	0.3	4.0	0.9648
More medications	0.6	0.2	2.5	0.5040
Smoker	1.7	0.4	7.1	0.4898
Diabetes	1.1	0.1	9.7	0.9278
Periodontitis history	1.1	0.3	4.6	0.9154
Shorter implant	5.8	1.3	26.4	**0**.**0230**
Narrower implant diameter	1.0	0.2	5.0	0.9790
Posterior implant*	6.1	0.3	111.3	0.2192
Maxilla	5.6	0.7	47.6	0.1113
Healing method (open)*	0.2	0.0	3.4	0.2535
Bone augmentation	5.5	1.4	21.7	**0**.**0174**
Lower torque	1.0	0.3	4.2	1.000

Significant *p*-values are marked in bold; *Because of non-convergence of the mixed model, a Firth-corrected logistic regression model was used instead to obtain estimates of odds ratios

## Discussion

In the absence of intimate bone-to-implant connection, osseointegration fails to occur, and fibrous scar tissue occurs instead. The results of this study suggest that implants placed in a practice-based setting heal successfully. With healing of approximately 95% for all implants placed, range of early failures was as expected from previous studies; however, the incidence of failure is somewhat toward the upper margin [10–18]. A large variety of patient-related and implant-related conditions can prevent successful osseointegration [10–18]. With regard to patient lifestyle, smoking is regarded as the primary risk factor for the failure of osseointegration [10–13]. Compromised blood circulation in the capillary border zone can lead to insufficient bone healing. Interestingly, this association was not identified in our study, and other recent studies have also failed to identify the use of tobacco as affecting early or late implant failure [15, 23]. It might also be speculated that the quantity of cigarettes smoked is associated with bone healing, as stated in a previous study [12]; this variable was not assessed in our study. Assuming a distribution of smokers and non-smokers similar to that recorded in our study (23.7% smokers and 76.3% non-smokers), it would be necessary to analyze results from 3169 implants to reveal a statistically significant difference in healing (*α* = 0.05; *β* = 0.8; calculation based on the chi-squared test). In addition to smoking, medical conditions such as diabetes mellitus and a history of periodontal disease are linked to poorer osseointegration [10–13]. Diabetes is a metabolic disease affecting blood circulation and, therefore, wound healing. In this study, however, no association was observed between the presence of diabetes and osseointegration; this might be because participants were compliant with anti-diabetic medication [24]. Some studies have found an association between periodontal disease and early implant failure [10–12]. In our study, participants with a history of periodontitis underwent periodontitis therapy before the implants were placed, and a history of periodontitis did not seem to be an affecting factor; this is also corroborated by previous research [25].

When the implant site (implant-related factors) has been evaluated, several studies have found that limited bone quality and quantity both have a substantial effect on the success of dental implants [11, 12]. Other studies have also revealed that implants placed in the posterior maxilla, especially, tend to be prone to early failure [12, 15, 16]. In this region, augmentation procedures (for example, sinus-floor elevation) are frequently needed to enable implant placement. In the literature, a greater incidence of failure is reported for this approach [13, 15, 17]. Similarly, our study showed that the use of augmentation procedures was associated with a greater risk of early implant failure. Grafting is, however, also used for the mandible in cases of low residual bone height or width. Moreover, when an implant is placed combined with simultaneous augmentation, the outcome is not consistently predictable at the moment of implant placement [26, 27]. For some augmentative measures there seems to be no difference with respect to clinical outcome between simultaneous augmentation and a staged approach (sinus floor elevation) [26]; however, i.e. in vertical / horizontal allogenic grafting a delayed approach offers the advantage to estimate bone and tissue formation prior to implantation [27]. In bivariate analysis, the incidence of implant failure tended to differ between implants placed in anterior and posterior sites, with more posterior failures. These trends are, however, in the same direction (insufficient bone, achieving a safe distance from the alveolar nerve). Interestingly, the use of short (< 10 mm) implants also resulted in a greater risk of implant failure. Because short implants can be used to avoid augmentation procedures, they are likely to be used when bone height is insufficient. In the literature, the greater risk of failure for short implants is a matter of controversy. Some studies have shown that length affects the healing of implants [14, 18]. Longer implants have a larger contact surface with the bone and have greater stability in sites with compromised bone quality in particular. This increased the likelihood of bone cells growing into the surface. Other studies, however, have either found no greater risk for short implants [11, 15] or a greater risk of failure solely for specific sites (maxilla) or implant type (machined surface) [16]. Another issue requiring discussion is that our study detected no associations between healing method (open or closed) and the torque applied while placing the implants. This partly contrasts with literature reports in which primary stability, for example, was presented as a factor affecting implant success [28] others, however, obtained the opposite result [29]. In our study, a torque of at least 20 Ncm was achieved; this is indicative of consistently acceptable primary stability. Moreover, it can be assumed that the surgeon responded to less implant stability at time of placement by allowing prolonged healing times. Conversely, some implants placed with an even higher torque and time of placement were immediately loaded. These implants consequently reduced the mean healing time in the study (approximately 5 months in the maxilla and 4.6 months in the mandible). However, none of the implants that were loaded immediately failed in this study. When comparing the healing times in this study with those in the literature, the average recommended healing time is 6 months in the maxilla and 3 months in the mandible, which should be prolonged in the case of augmentation. The mean healing time in this study is thus slightly shorter for implants placed in the maxilla and slightly longer for those placed in the mandible.

### Strengths and weaknesses of the study

When interpreting and generalizing the study outcome, it should be kept in mind that the study was retrospective in nature and the sample size rather small. Because of the well-defined surgical protocol and structured documentation, however, the practice data used seem to be comparable in quality with those from prospective approaches. Nevertheless, when target variables and statistical approaches are selected retrospectively for existing data, a certain lack of clarity might be expected; for smokers, for example, the quantity of cigarettes smoked per day or the exact periodontal diagnosis was not available. Another bias caused by the retrospective study design can be found in ‘non-responding participants’; controlling for drop-outs is more transparent in prospective studies. In fact, our study achieved a high response rate of approx. 90%, which means that the bias is lower than that usually accepted in retrospective studies. With regard to bias, it should perhaps also be kept in mind that the professional experience of dentists working in general practice will vary, which could affect the success or failure of surgical procedures. In this study, the two dentists had amassed professional experience of between two and 9 years.

## Conclusions

Within the limitations of this study, early failure rates of dental implants placed in the dental practice are similar to those seen in previous studies, however, rates are also comparable to failure rates reported from university studies, eventhough somewhat toward the upper margin. The use of augmentation procedures and implants shorter than 10 mm seem to be associated with early implant failure. Health-service research with larger samples is encouraged to verify these associations.

The outcome of this study can help practitioners to estimate the probable success of their dental implants and to assess the suitability of surgical implant procedures. For sites with reduced bone quantity, the use of shorter implants might be an alternative to augmentative approaches; however, patients should be informed about the lower early success rates of both strategies.

## Abbreviations

%: percent
Approx: approximately
CI: confidence interval
D1: dentist 1
D2: dentist 2
E. g.: for example
Mm: millimetre
Ncm: Newtoncentimeter
SD: standard deviation

## References

[1] Kassebaum NJ, Bernabé E, Dahiya M, Bhandari B, Murray CJL, Marcenes W (2014). Global burden of severe tooth loss: a systematic review and meta-analysis. J Dent Res.

[2] Haag DG, Peres KG, Balasubramanian M, Brennan DS (2017). Oral conditions and health- related quality of life: a systematic review. J Dent Res.

[3] John MT, Koepsell TD, Hujoel P, Diana LM, Linda L, Wolfgang M (2014). Demographic factors, denture status and oral health-related quality of life. Community Dent Oral Epidemiol.

[4] Pjetursson BE, Lang NP (2008). Prosthetic treatment planning on the basis of scientific evidence. J Oral Rehabil.

[5] Dhingra K (2012). Oral rehabilitation considerations for partially edentulous periodontal patients. J Prosthodont.

[6] Herrero-Climent M, Lázaro P, Vicente Rios J, Lluch S, Marque S, Mart ı M G (2013). Gil FJ Influence of acid-etching after grit-blasted on osseointegration of titanium dental implants: in vitro and in vivo studies. J Mater Sci Mater Med.

[7] Rupp F., Liang L., Geis-Gerstorfer J., Scheideler L., Hüttig F. (2018). Surface characteristics of dental implants: A review. Dental Materials.

[8] Ketabi M, Deporter D, Atenafu EG (2016). A systematic review of outcomes following immediate molar implant placement based on recently published studies. Clin Implant Dent Relat Res.

[9] Abou-Ayash S, Strasding M, Rücker G, Att W (2017). Impact of prosthetic material on mid- and long-term outcome of dental implants supporting single crowns and fixed partial dentures: a systematic review and meta-analysis. Eur J Oral Implantol.

[10] Chrcanovic BR, Kisch J, Albrektsson T, Wennerberg A (2016). Factors influencing early dental implant failure. J Dent Res.

[11] Grisar K, Sinha D, Schoenaers J, Titiaan D, Constantinus P (2017). Retrospective analysis of dental implants placed between 2012 and 2014: indications, risk factors, and early survival. Int J Oral Maxillofac Implants.

[12] van Steenberghe D, Jacobs R, Desnyder M, Maffei G, Quirynen M (2002). The relative impact of local and endogenous patient-related factors on implant failure up to the abutment stage. Clin Implant Dent Relat Res.

[13] Antoun H, Karouni M, Abitbol J, Zouiten O, Jemt T (2017). A retrospective study on 1592 consecutively performed operations in one private referral clinic. Part I: early inflammation and early implant failures. Clin Implant Dent Relat Res.

[14] Olate S, Lyrio MC, de Moraes M, Mazzonetto R, Moreira RWF (2010). Influence of diameter and length of implant on early dental implant failure. J Oral Maxillofac Surg.

[15] Borba M, Deluiz D, Lourenço EJV, Oliveira L, Tannure PN (2017). Risk factors for implant failure: a retrospective study in an educational institution using GEE analyses. Braz Oral Res.

[16] Pommer B, Frantal S, Willer J, Posch M, Watzek G, Tepper G (2011). Impact of dental implant length on early failure rates: a meta-analysis of observational studies. J Clin Periodontol.

[17] Shi JY, Gu YX, Zhuang LF, Lai H-C (2016). Survival of implants using the Osteotome technique with or without grafting in the posterior maxilla: a systematic review. Int J Oral Maxillofac Implants.

[18] Manzano G, Montero J, Martín-Vallejo J, Del Fabbro M, Bravo M, Testori T (2016). Risk factors in early implant failure: a meta-analysis. Implant Dent.

[19] Balevi B (2014). Patients who received preoperative antibiotics showed fewer early implant failures. J Am Dent Assoc.

[20] Papaspyridakos P (2015). Implant success rates for single crowns and fixed partial dentures in general dental practices may be lower than those achieved in well-controlled university or specialty settings. J Evid Based Dent Pract.

[21] Noelken R, Kunkel M, Jung BA (2014). Immediate nonfunctional loading of NobelPerfect implants in the anterior dental arch in private practice--5-year data. Clin Implant Dent Relat Res.

[22] Jemt T (2017). A retro-prospective effectiveness study on 3448 implant operations at one referral clinic: A multifactorial analysis. Part I: Clinical factors associated to early implant failures. Clin Implant Dent Relat Res.

[23] Zupnik J, Kim SW, Ravens D, Karimbux N, Guze K (2011). Factors associated with dental implant survival: a 4-year retrospective analysis. J Periodontol.

[24] Balshi TJ, Wolfinger GJ (1999). Dental implants in the diabetic patient: a retrospective study. Implant Dent.

[25] Gianserra R, Cavalcanti R, Oreglia F, Massimo Francesco M, Marco E (2010). Outcome of dental implants in patients with and without a history of periodontitis: a 5-year pragmatic multicentre retrospective cohort study of 1727 patients. Eur J Oral Implantol.

[26] Wallace SS, Froum SJ (2003). Effect of maxillary sinus augmentation on the survival of endosseous dental implants. A systematic review. Ann Periodontol.

[27] Draenert FG, Kämmerer PW, Berthold M, Neff A (2016). Complications with allogeneic, cancellous bone blocks in vertical alveolar ridge augmentation: prospective clinical case study and review of the literature. Oral Surg Oral Med Oral Pathol Oral Radiol.

[28] Ottoni JM, Oliveira ZF, Mansini R (2005). Correlation between placement torque and survival of single-tooth implants. Int J Oral Maxillofac Implants.

[29] Berardini M, Trisi P, Sinjari B, Rutjes AW, Caputi S (2016). The effects of high insertion torque versus low insertion torque on marginal bone resorption and implant failure rates: a systematic review with meta-analyses. Implant Dent.

